# Prevention and Screening for Cardiometabolic Disease Following Hypertensive Disorders in Pregnancy in Low-Resource Settings: A Systematic Review and Delphi Study

**DOI:** 10.5334/gh.1195

**Published:** 2023-04-25

**Authors:** Salisu Mohammed Ishaku, Kwame Adu-Bonsaffoh, Natasha Housseine, Roberta Lamptey, Arie Franx, Diederick Grobbee, Charlotte E. Warren, Joyce L. Browne

**Affiliations:** 1 Julius Global Health, Julius Center for Health Science and Primary Care, University Medical Center, Utrecht, NL; 2 Julius Global Health, Julius Center for Health Sciences and Primary Care, University Medical Center Utrecht, Utrecht University, the Netherlands; 3 Department of Obstetrics and Gynecology, University of Ghana Medical School, Korle Bu Teaching Hospital Accra, GH; 4 Medical College, Aga Khan University, Dar es Salaam, TZ; 5 Department of Family Health, Korle Bu Teaching Hospital and Department of Community Health, GH; 6 Department of Obstetrics and Gynecology, Erasmus Medical Center, Rotterdam, NL; 7 Population Council, Washington, DC, US

**Keywords:** Hypertensive disorders in pregnancy, risks of future cardiometabolic diseases, prevention and screening strategies

## Abstract

Hypertensive disorders in pregnancy (HDP) and cardiometabolic and kidney diseases are rising in low- and middle-income countries (LMICs). While HDP are risk factors for cardiometabolic and kidney diseases, cost-effective, scalable strategies for screening and prevention in women with a history of HDP are lacking. Existing guidelines and recommendations require adaptation to LMIC settings. This article aims to generate consensus-based recommendations for the prevention and screening of cardiometabolic and kidney diseases tailored for implementation in LMICs.

We conducted a systematic review of guidelines and recommendations for prevention and screening strategies for cardiometabolic and chronic kidney diseases following HDP. We searched PubMed/Medline, Embase and Cochrane Library for relevant articles and guidelines published from 2010 to 2021 from both high-income countries (HICs) and LMICs. No other filters were applied. References of included articles were also assessed for eligibility. Findings were synthesized narratively. The summary of guiding recommendations was subjected to two rounds of Delphi consensus surveys with experts experienced in LMIC settings.

Fifty-four articles and 9 guidelines were identified, of which 25 were included. Thirty-five clinical recommendations were synthesized from these and classified into six domains: identification of women with HDP (4 recommendations), timing of first counseling and provision of health education (2 recommendations), structure and care setting (12 recommendations), information and communication needs (5 recommendations), cardiometabolic biomarkers (8 recommendations) and biomarkers thresholds (4 recommendations). The Delphi panel reached consensus on 33 final recommendations.

These recommendations for health workers in LMICs provide practical and scalable approaches for effective screening and prevention of cardiometabolic disease following HDP. Monitoring and evaluation of implementation of these recommendations provide opportunities for reducing the escalating burden of noncommunicable diseases in LMICs.

## Introduction

Evidence of associations between prior hypertensive disorders in pregnancy (HDP, which includes preeclampsia, chronic hypertension in pregnancy and gestational hypertension) [1] and long-term risk of cardiometabolic and chronic kidney diseases is mounting and getting stronger. The occurrence of persistent hypertension and/or new onset cardiovascular diseases in women with prior HDP have been reported in high-income countries (HICs) [2,3] and low- and middle-income countries (LMICs) [4,5,6,7,8,9]. This is similarly the case for metabolic syndrome and chronic kidney diseases [10,11,12,13,14,15]. In recent years, a growing number of scientific articles, guidelines and recommendations on effective prevention and screening strategies for cardiometabolic and chronic kidney diseases following HDP have been published [16,17,18,19]. However, these recommendations were developed within the standards of practices from HICs and often may not be feasible in LMIC settings due to their different contexts and health system dynamics [20]. As a result, widespread adoption, implementation and evaluation of prevention and screening strategies for women with a history of HDP have not been achieved in LMIC settings. This requires urgent attention, especially in LMIC settings, where the prevalence of both the HDP and noncommunicable diseases are increasing [21,22]. Therefore, this study aimed to generate simple, user-friendly, practicable and effective recommendations for prevention and early detection of cardiometabolic and chronic kidney diseases following HDP in LMIC.

## Methodology

### Study design

A systematic review was conducted, followed by a Delphi consensus survey.

**Systematic review**

For the systematic review, we searched the following electronic bibliographic databases: PubMed/MEDLINE (MeSH terms and tiab searches), Embase (Emtree) and Cochrane Library for relevant articles and guidelines using terms related to hypertensive disorders in pregnancy, cardiovascular disease, metabolic syndrome and chronic kidney disease. Appendix I shows the search strategy used for PubMed, which was slightly modified to suit other databases. Searches were restricted to publications in English and articles published within the last 10 years (2010–2021) to reflect current practice evidence. No other restrictions were applied. The review was registered in PROSPERO (Ref no: CRD42021279362). The search was conducted with the support of a specialized librarian. References of included articles were also assessed for eligibility (snowballing). Reports retrieved from different search engines were combined in Endnote, and duplicates were manually removed. Titles and abstracts of studies retrieved from the search strategy were screened, based on the inclusion/exclusion criteria, by two independent reviewers (SMI & KA) using Rayyan software (https://rayyan.ai/). Conflict between the two reviewers was jointly resolved, and, if necessary, a third member of the team was consulted. Full texts of eligible articles were retrieved and assessed by one member of the review team (SMI). Data extraction was conducted through a standardized data extraction form that was designed based on predetermined variables (authorship, years of publication, country of publication, titles and guiding recommendations). Data extraction was performed by a single reviewer who was not blinded for journal or author details. Box 1 provides operational definitions for cardiometabolic and chronic kidney diseases used in this article.

**Box I Operational Definitions**

*Cardiovascular disease*: This included systemic hypertension (occurrence of any blood pressure measurement of ≥140 mm Hg systolic and/or ≥90 mm Hg diastolic after deliveries of HDP-complicated pregnancies), cerebrovascular accidents and any abnormal physiological or morphological changes affecting the heart that is reported to have occurred following pregnancies complicated by HDP.

*Chronic kidney disease*: Any reported decrease in estimated glomerular filtration rate of less than 60 mL/min/1.73m^2^ lasting for ≥ three months (or any acceptable indicator measure of chronic kidney disease) occurring following deliveries of pregnancies complicated by HDP.

*Metabolic syndrome*: All reported cases of metabolic syndrome as defined by the following authorities:

- The World Health Organization (WHO)—Hyperinsulinemia (the upper fourth of the fasting insulin level among nondiabetic subjects) or hyperglycemia (fasting glucose ≥110 mg/dl) in addition to at least two of the following: waist girth ≥94 cm; dyslipidemia (triglycerides ≥150 mg/dl or HDL cholesterol ≥40 mg/dl); blood pressure ≥140/90 mm Hg; or taking blood pressure medication.
- The International Diabetes Federation consensus statement—The presence of body mass index (BMI) >30kg/m^2^ with any two of the following: triglycerides ≥1.7 mmol/L; high-density lipoprotein <1.29 mmol/L; hypertension (systolic BP ≥130 or diastolic BP ≥85 mmHg); or fasting blood glucose ≥100 mg/dL or 5.6 mmol/L.
- The National Cholesterol Education Program–NCEP ATP III—The presence of any three of the following: hypertension ≥130/85 mm Hg; fasting plasma glucose ≥6.1 mmol/L; fasting plasma triglycerides ≥1.69 mmol/L; fasting HDL cholesterol <1.29 mmol; or subjects were receiving treatment for their condition and had a waist circumference >88 cm.

### Delphi survey

Following the initial summary of the systematically derived evidence on global practices for prevention and screening of women with prior HDP, the guiding recommendations were subjected to Delphi surveys through two rounds of reviews and scoring by experts with experience working in LMICs, such that only those with consensus on promising effectiveness and thought to be low cost, feasible and practicable in LIMCs were recommended as practice guidelines.

The rounds of online Delphi surveys were conducted among obstetricians, midwives, endocrinology physicians and nephrologists according to predefined objectives and criteria for experts panel selection. The surveys took place in March and April 2022. Prior to the surveys, ethical review exemption was sought from the medical ethics committee of the University Medical Center Utrecht, Utrecht University, the Netherlands.

To identify all relevant survey participants, we used both targeted searching and listing of known experts and disseminated messages across professional organizations to request for participation. A formal email invitation was sent to all relevant experts preidentified and those identified through affiliations with professional bodies. The Delphi survey was developed in SurveyMonkey and pilot tested by members of the research team to enable subsequent adjustments. Two rounds of surveys were conducted, each with an average closing date of three weeks. Stakeholders who did not participate in the first round were not invited for second round.

The stakeholders were asked to make recommendations that were deemed feasible, affordable and safe for medical practitioners in low-resource settings given that both human and material resources are limited and/or not widely available or affordable in these settings. Based on the summarized evidence from the systematic review, a questionnaire was developed (Appendix I), and stakeholders were asked to quantify their level of support for potential recommendations via a five-item Likert scale (1, strongly disagree; 2, disagree; 3, neutral; 4, agree; 5, strongly agree). Space was provided for free-text feedback when indicated. Survey responses were analyzed automatically on the SurveyMonkey platform. For each, outcome, frequencies and percentages of level of agreement were calculated per stakeholder response.

Consensus was defined as agreements among stakeholders of at least 70% scoring an item as “agree/strongly agree” and less than 15% scoring it as “disagree/strongly disagree.” Exclusion of items required at least 70% of stakeholders scoring the item as “disagree/strongly disagree” and less than 15% scoring it as “agree/strongly agree.” Items that did not meet these criteria were classified as “no consensus.” If consensus was reached, participants were informed, and the outcome was left out from the next round. Outcomes that nearly reached consensus were discussed by the study team for a final decision.

## Results

### Systematic review

Of 63 articles and guidelines retrieved from the search, 25 were found to be relevant and included for the study. Characteristics of included articles are presented in Table 1. The recommendations in these included articles were reviewed and summarized by the research team and resulted in 35 guiding statements. These were used in the questionnaire of the Delphi survey (see Appendix I). The guiding recommendations were classified into six domains: identification of women with HDP (4 recommendations), timing of first counseling and provision of health education (2 recommendations), structure and care setting (12 recommendations), information and communication needs (5 recommendations), cardiometabolic biomarkers (8 recommendations) and biomarkers thresholds (4 recommendations).

**Table 1:** Results of systematic review displayed by authorship, year of publication, article titles and recommendations for prevention and screening of cardiometabolic and chronic kidney diseases in women with prior hypertensive disorders in pregnancy (HDP, preeclampsia, gestational hypertension and chronic hypertension in pregnancy).


PREVENTION AND SCREENING OF CARDIOVASCULAR DISEASES AND METABOLIC SYNDROME FOLLOWING HYPERTENSIVE DISORDERS IN PREGNANCY				

AUTHORS	YEAR	COUNTRY	TITLE	RECOMMENDATIONS

Deborah B. Ehrenthal, Janet M. Catov	2013	United States	Importance of engaging obstetrician/gynecologists in cardiovascular disease prevention	Obstetricians and gynecologists should routinely counsel women on their risk for cardiometabolic disorders, as they have the best opportunities for doing so.

Emely J. Jones, Teri L. Hernadez, Joyce K. Edmonds, Erin P. Ferranti	2019	United States	Continued disparities in postpartum follow-up and screening among women with gestational diabetes and hypertensive disorders of pregnancy: A systematic review	Obstetricians and gynecologists should routinely counsel women on their risk for cardiometabolic disorders, as they have the best opportunities for doing so.

Anouk Bokslag, Wietske Hermes, Christianne J. M. de Groot, Pim W. Teunissen	2016	Netherlands	Reduction of cardiovascular risk after preeclampsia: The role of framing and perceived probability in modifying behavior	Counseling women on behavior modification should express the risk of cardiometabolic disorders as probability scores, expressed as chance of developing the disease condition.

Fitriana Murriya Ekawati, Sharon Licqurish, Jane Gunn, Shaun Brennecke, Phyllis Lau	2021	Indonesia	Hypertensive disorders of pregnancy (HDP) management pathways: Results of a Delphi survey to contextualize international recommendations for Indonesian primary care settings	Counseling on contraception should be done. Annual monitoring should be done.

Tessa E. R. Gillon, Anouk Pels, Peter von Dadelszen, Karen MacDonell, Laura A. Magee	2014	Multinational	Hypertensive disorders in pregnancy: A systematic review of international clinical practice guidelines	Postpartum lifestyle counseling for BMI should be done, especially in obese women.

T. Katrien J. Groenhof, Bas B. van Rijn, Arie Franx, Jeanine E. Roeters van Lennep, Michiel L. Bots, A. Titia Lely	2017	Multicountry	Preventing cardiovascular disease after hypertensive disorders of pregnancy: Searching for the how and when	It is not possible to identify a time point to commence screening for cardiovascular risk factors in women after an HDP. Women with HDP should be screened in a stepwise fashion (taking into account classical risk factors, HDP phenotype, genetics, etc.) starting at a young age. The first appropriate approach for the management of cardiovascular risks should be lifestyle modification.

T. Katrien J. Groenhof, Gerbrand A. Zoet, Arie Franx, Ron T. Gansevoort, Michiel L. Bots, Henk Groen, A. Titia Lely	2019	Netherlands	Trajectory of cardiovascular risk factors after hypertensive disorders of pregnancy: An argument for follow-up	Cardiovascular screening of women with a history of HDP should commence within the fourth decades of life.

Alisse Hauspurg, Malamo E. Countouris, Janet M. Catov	2019	United States	Hypertensive disorders of pregnancy and future maternal health: How can the evidence guide postpartum management?	Where feasible, blood pressure (BP) within the first year postpartum should be monitored, preferably by ambulatory BP monitoring (ABPM) or home BP monitoring, to improve hypertension detection rate. Health care providers should carry out postpartum cardiovascular disease (CVD) risk counseling and screening, as women are more motivated during this period. All maternity centers should formulate dedicated guidelines for women with HDP on their continuity of care from obstetricians/midwives, primary care physicians or specialists, as appropriate. Postpartum care counseling should be delivered within a trauma-informed model, as women with post-traumatic experience are less likely to return to health facilities for regular monitoring. Where feasible, women with HDP should be reviewed within a multidisciplinary clinic involving obstetricians/midwives, primary care physicians and cardiologists to reduce inequities in health. Where practicable, a dedicated postpartum clinic for HDP should be established to enhance and facilitate transition of care and to provide a window of opportunity to focus on improving cardiometabolic health, primary prevention of CVD and counseling on risk factor modification. The inclusion and utilization of best practice alerts in electronic medical records should be adopted to facilitate risk identification and improve follow-up. In women with HDP, regular BP and cholesterol screening should be more frequent and should start as soon as one year postpartum. Women with a history of HDP should be counseled to follow diets, such as the DASH diet, that are low in salt and animal fat and include a majority of fruits and vegetables and more plant-based protein. Women with HDP should be advised to do moderate exercise for at least 30 minutes a day, five times per week. Women who smoke cigarettes should be counseled on smoking cessation.

American Heart Association	2011	United States	Effectiveness-based guidelines for the prevention of cardiovascular disease in women—2011 update: A guideline from the American Heart Association	Women with a history of hypertensive disorders of pregnancy should have careful screening, monitoring, and control of other CVD risk factors, such as hypertension, dyslipidemia and diabetes.

Maria Carolina Gongora, Garima Sharma, Eugene Yang	2018	United States	Hypertension during pregnancy and after delivery: Management, cardiovascular outcomes and future directions	A comprehensive pregnancy history tool for CVD risk assessment should be developed to enable elucidation of nontraditional CVD risk factors.

Canadian Hypertensive Disorders of Pregnancy Working Group/Society of Obstetricians and Gynecologists of Canada	2014	Canada	Diagnosis, evaluation, and management of the hypertensive disorders of pregnancy: Executive summary	Women who are overweight should be encouraged to attain a healthy body mass index (BMI) to decrease risk in future pregnancy (II-2A) and for long-term health. Women with preexisting hypertension or persistent postpartum hypertension should undergo the following investigations (if not done previously) at least six weeks postpartum: urinalysis; serum sodium, potassium and creatinine; fasting glucose; fasting lipid profile; and standard 12-lead electrocardiography. Women who are normotensive but have had a hypertensive disorder of pregnancy may benefit from assessment of traditional cardiovascular risk markers. All women who have had a hypertensive disorder of pregnancy should pursue a healthy diet and lifestyle.

Susanne H. E. Luitjes	2013	Netherlands	Implementation of Dutch guidelines on hypertensive disorders of pregnancy	Antenatal, intrapartum and postpartum care for patients with chronic hypertension should be provided by gynecologists.

Roopa Malik, Viral Kumar	2017	India	Hypertension in pregnancy	Lifestyle modification (maintenance of a healthy weight, increased physical activity and smoking cessation) are recommended. In women with history of recurrent and early onset preeclampsia, yearly BP, fasting blood glucose and BMI should be done.

J. Moodley, P. Soma-Pillay, E. Buchmann, R. C. Pattinson	2019	South Africa	South African 2019 national guidelines for hypertensive disorders in pregnancy	Depending on where the woman lives, a follow-up at a hospital or community health center should take place after one week. A three-monthly follow-up is recommended. Psychological health counseling and support should be provided.

Elizabeth Phipps, Devika Prasanna, Wunnie Brima, Belinda Jim	2016	United States	Preeclampsia: Updates in pathogenesis, definitions, and guidelines	A cardiovascular profile, including yearly assessment of BP, lipids, fasting blood glucose, and BMI, in women with a history of preterm preeclampsia or recurrent preeclampsia should be done.

Gitte Bro Schmidt, Martin Christensen, Ulla Breth Knudsen	2017	Denmark	Preeclampsia and later cardiovascular disease—What do national guidelines recommend?	Inform about risk of increase in BP and risk of CVD later in life Healthy diet, exercise, smoking cessation and healthy BMI Postpartum CVD risk screening Annual BP assessment and blood tests/other CVD risk factors Later life follow-up When to start screening: 3–5 months after index pregnancy; 10 years after PE; around the age of 50 Three months after delivery, screening for hypertensive disease Long-term monitoring of cardiovascular and metabolic risk factors recommended to patients after severe preeclampsia

Malia S. Q. Murphy, Graeme N. Smith	2016	Canada	Pre-eclampsia and cardiovascular disease risk assessment in women	The development of structured postpartum cardiovascular screening programs for women after HDP is essential. The timing and nature of postpartum intervention plays a key role in women’s receptivity to lifestyle change. Women are generally highly motivated to lower their CVD risk if interventions are available and accessible. Professional counseling and involvement are key to success. A combination of educational, nutritional and physical activity resources to target an individual’s specific needs is needed. Promotion of a healthy lifestyle in conjunction with close monitoring and treatment of individual CVD risk factors is likely to be the most promising approach to CVD prevention in young at-risk women.

Ellen W. Seely, Ann C. Celi, Jaimie Chausmer, Cornelia Graves, Sarah Kilpatrick, Jacinda M. Nicklas, Mary L. Rosser, Kathryn M. Rexrode, Jennifer J. Stuart, Eleni Tsigas, Jennifer Voelker, Carolyn Zelop, Janet W. Rich-Edwards	2020	United States	Cardiovascular health after preeclampsia: Patient and provider perspective	Increased awareness and action to prevent CVD after preeclampsia A clinician checklist to ensure communication of CVD risks (patients’ perspective) Enhanced training for clinicians on the link between preeclampsia and CVD (patients’ perspective) A postdelivery appointment with a clinician knowledgeable about the link between preeclampsia and CVD risk (patients’ perspective) Clinical programs primarily to serve patients in the first postpartum year, bridging obstetrical and primary care (patients’ perspective) CVD risk modification with periodic BP, weight, lipids and diabetes screening (patients’ perspective) Integrated efforts of patients, caregivers, researchers and national organizations are needed to improve CVD prevention after preeclampsia. Risk modification programs could start between six weeks and six months postpartum and could be delivered online, via a mobile device or peer-to-peer support in a safe community of shared experience (patients’ perspectives). The clinical postpartum program could start within the first year postpartum or years after. Follow-up should be limited to only women with HDP OR include women with other obstetrics indicators, such as preterm delivery, intrauterine growth restriction and gestational diabetes. Women should be educated and empowered to stop smoking, adopt a heart healthy diet and engage in physical activity to lower BP, weight, glucose and cholesterol.

National Institute for Health and Care Excellence	2019	United Kingdom	Hypertension in pregnancy: Diagnosis and management	Women who had HDP should be advised that this is associated with increased risk of hypertension and cardiovascular disease later in life. Women can reduce their risk of cardiovascular disease and hypertension by avoiding smoking and maintaining a healthy lifestyle and healthy weight.

Laura Benschop, Johannes J Duvekot, Jeanine E. Roeters van Lennep	2019	Netherlands	Future risk of cardiovascular disease risk factors and events in women after a hypertensive disorder of pregnancy	All women with HDP should be informed of their increased risk of metabolic syndrome and cardiovascular diseases in later life. Women with HDP (especially the overweight—BMI ≥25kg/m^2^) should be encouraged to adopt a healthy diet (fruits and vegetables) and healthy lifestyle (physical activity, no smoking, moderate alcohol, etc.). Screening for cardiometabolic risk factors should commence during the six- to eight-week postpartum visit (minus lipids profile) and annually thereafter (to include lipids profile).

Lauren Rockliffe, Sarah Peters, Alexander E. P. Heazell, Debbie M. Smitha	2021	United Kingdom	Understanding pregnancy as a teachable moment for behavior change: A comparison of the COM-B and teachable moments models	Counseling and health education information should be provided during pregnancy, as pregnancy provides a better teachable moment for the adoption of healthy living.

D. M. Folk	2018	United States	Hypertensive disorders of Pregnancy: Overview and current recommendations	If counseling was not provided during pregnancy, the next best opportunities should be either in the immediate postpartum period before discharge or during the two-week postpartum review. Women who had preeclampsia should be counseled postpartum about the risks of cardiovascular diseases and be referred to a primary care provider or cardiologist for continued follow-up care.

S. A. Lowe, L. Bowyer, K. Lust, L. P. McMahon, M. Morton, R. A. North, M. Paech, J. M. Said	2014	Australia and New Zealand	Society of Obstetric Medicine of Australia and New Zealand (SOMANZ): Guideline for the management of hypertensive disorders of pregnancy 2014	Women who have had preeclampsia should be counseled that they will benefit from avoiding smoking, maintaining a healthy weight, exercising regularly and eating a healthy diet. All women with previous preeclampsia or hypertension in pregnancy should have an annual BP check and regular (five yearly or more frequent if indicated) assessment of other cardiovascular risk factors, including serum lipids and blood glucose.

**PREVENTION AND SCREENING OF CHRONIC KIDNEY DISEASES**				

German Association of Obstetrics and Gynecology	2010	Germany	Diagnostik und Therapie hypertensiver Schwangerschaftserkrankungen: Arbeitsgemeinschaft Schwangerschaftshochdruck/Gestose AWMF	Three months after delivery, screening for renal disease should be done. Long-term monitoring of renal risk factors should be recommended to patients after severe preeclampsia.

National Institute for Health and Care Excellence	2019	United Kingdom	Hypertension in pregnancy: Diagnosis and management	Women with HDP should have their urine protein estimated six to eight weeks postpartum. If there is no proteinuria or hypertension during this review, no further follow-up is necessary.

Canadian Hypertensive Disorders of Pregnancy Working Group/Society of Obstetricians and Gynecologists of Canada	2014	Canada	Diagnosis, evaluation, and management of the hypertensive disorders of pregnancy: Executive summary	Women with a history of severe preeclampsia (particularly those who presented or delivered before 34 weeks’ gestation) should be screened for preexisting hypertension and underlying renal disease. Referral for internal medicine or nephrology consultation (by telephone if necessary) should be considered for women with (i) postpartum hypertension that is difficult to control or (ii) women who had preeclampsia and have at three to six months postpartum either ongoing proteinuria, decreased estimated glomerular filtration rate (eGFR) (<60 mL/min) or another indication of renal disease, such as abnormal urinary sediment.


### Delphi survey

In the first round of the survey, a total of 104 invitations were sent to experts with significant experience on practice in LMICs (comprising 70 practicing obstetricians (67.3%), 11 FIGO representatives (10.6%), 13 midwives (12.5%) and 16 journal editors (15.4%)), out of which 3 (2.9%) opted out and 18 (17.3%) did not respond, giving a response rate of 79.8% (n = 83 invitees: 47 practicing obstetricians, 10 FIGO representatives, 11 midwives and 15 journal editors). In the second round, 83 invitations were sent with an 88% response rate (n = 73 invitees: 45 (61.6%) practicing obstetricians, 5 (6.8%) FIGO representatives, 10 midwives (14.3%) and 13 (17.8%) journal editors). The respondents were from Bangladesh, Brazil, Chile, China, Colombia, Denmark, Egypt, Ethiopia, India, Iraq, Kenya, Lebanon, Malawi, Malaysia, Namibia, Netherlands, Nigeria, Panama, Philippines, Portugal, South Africa, Sweden, Tanzania, Thailand, Uganda, United Kingdom, United States of America, Uruguay, Venezuela and Zimbabwe.

The summary of the survey outcomes by round of survey in which a consensus was reached on whether to include or exclude a recommendation is presented in Appendix II. On identification of women with HDP based on the ISSHP definition, consensus was reached among 80% (n = 66) of the surveyed participants during the first round of the survey, while for the definitions of gestational hypertension, chronic hypertension in pregnancy and preeclampsia, consensus was achieved in the second round (n = 59, 78%). As for the best timing to initiate counseling and health education, all the two recommendations received consensus in the first round with 80% (n = 66) and 76% (n = 63) approvals, respectively (*recommendations*: counseling to commence as early as possible during the antenatal period *and* counseling and health/education to commence in the immediate postpartum period if prenatal counseling/education was not provided).

For the structure and setting of care, 11 of the 12 guiding recommendations got a consensus approval during the first survey round, with rating ranging from 75% (*recommendation*: women with other nontraditional risk factors for cardiometabolic diseases should also be monitored postpartum) to 98% (*recommendation*: counseling should be performed at facilities that women can access and by any available trained health care provider, regardless of their specialty). One recommendation was eliminated in the first round, as only 36% of the surveyed participants approved (*recommendation*: postpartum care counseling should be delivered within a trauma-informed model).

Regarding counseling needs of women diagnosed with HDP, two recommendations had consensus approval in the first round (*recommendations*: all women with HDP should be informed of their increased risk of cardiometabolic and chronic kidney diseases in later life *and* counseling should be expressed as chances (%) of developing the disease condition), while the remaining three recommendations achieved approval consensus in the second round. In respect to the actual screening tests to be performed, of the eight recommendations subjected to the survey, seven received consensuses (six in the first round and one in the second round), while one recommendation was eliminated in the first round with only 34% approval (*recommendation*: women with HDP should have their urine protein (dipstick measurement or 24-hour urine protein estimation) estimated six to eight weeks postpartum; if there is no proteinuria or hypertension during this review, no further follow-up is necessary).

Finally, all proposed indicators of abnormal test results got consensus approval during the first survey phase. In total, 33 of the initial 35 guiding recommendations put forth by the research team received consensus approval and constitute the final guidelines recommended for prevention and screening of cardiometabolic and kidney diseases in women with prior HPD in LMICs (see Table 2).

**Table 2:** Recommended guiding practices for prevention and screening of cardiometabolic and kidney diseases in women with prior hypertensive disorders in pregnancy (HDP), for use in low- and middle-income countries (LMICs).


GUIDELINE NO.	IDENTIFYING WOMEN WITH HDP

1.	As recommended by the ISSHP, HDP should be classified as chronic hypertension in pregnancy, gestational hypertension and preeclampsia.

2.	Chronic hypertension in pregnancy should be diagnosed as any hypertension with onset before the index pregnancy or diagnosed within the first 20 weeks of the index pregnancy.

3.	Gestational hypertension should be defined as hypertension arising de novo after 20 weeks’ gestation in the absence of proteinuria and without biochemical or hematologic abnormalities.

4.	Preeclampsia should be diagnosed as de novo hypertension after 20 weeks’ gestation accompanied by proteinuria and/or evidence of maternal acute kidney injury, liver dysfunction, neurological features, hemolysis or thrombocytopenia or fetal growth restriction.

	**Timing of First Counseling/Health Education**

5.	Counseling on cardiometabolic risk following HDP should start early in pregnancy with the diagnosis of the condition.

6.	If counseling was not provided during the pregnancy, the next best opportunities should be either in the immediate postpartum period before discharge *or* during the two-week postpartum review.

	**Structure and Setting of Care**

7.	Counseling should be performed at facilities that women can access and by any available trained health care provider, regardless of their specialty.

8.	Where feasible, women with HDP should be reviewed within a multidisciplinary clinic involving obstetricians/midwives, primary care physicians, cardiologists and mental health experts.

9.	Obstetricians, midwives and maternity care providers should routinely counsel women with HDP on their risk for cardiometabolic and kidney disorders.

10.	Where practicable, a dedicated postpartum clinic for HDP be established to facilitate transition of care and to provide a window of opportunity to focus on improving cardiometabolic health, primary prevention of cardiovascular disease (CVD) and counseling on risk factor modification.

11.	The inclusion and utilization of best practice alerts in electronic medical records should be adopted to facilitate risk identification and improve follow-up.

12.	All maternity centers should formulate a dedicated guideline for women with HDP for their continuity of care from obstetricians/midwives, primary care physicians or specialists, as appropriate.

13.	All maternity centers should develop a comprehensive pregnancy history tool for CVD risk assessment to enable elucidation of nontraditional CVD risk factors (for example, gestational diabetic, intrauterine growth restriction and preterm delivery).

14.	Women with other nontraditional risk factors for cardiometabolic diseases, such as gestational diabetes, intrauterine growth restriction and preterm delivery, should also be counseled and monitored postpartum.

15.	Where feasible, the antenatal care card/folder/record should be modified to include a section on documentation of postpartum risk assessment and monitoring of long-term risks of chronic medical conditions associated HDP and other pregnancy complications.

16.	All health care providers of maternity services should be trained on the links between HDP and cardiometabolic and chronic kidney disorders.

17.	A health care provider checklist should be provided as a working tool to ensure detailed and balanced communication of cardiometabolic disease risks to patients with HDP.

	**Counseling Information Needs for women Identified with HDP**

18.	All women with HDP should be informed of their increased risk of cardiometabolic and chronic kidney diseases in later life.

19.	Counseling women on behavior modification should express the risk of cardiometabolic disorders as probability scores, expressed as chances (%) of developing the disease condition.

20.	Women with HDP (especially women who are overweight—BMI ≥25kg/m2) should be informed that postpartum lifestyles modification, as the first approach, substantially reduces the risk of cardiometabolic diseases in later life.

21.	Lifestyle modification should include adopting a healthy diet (all or any combination of consumption of fruits, vegetables, plant protein and oily fish *and* reduction or combination of any of diets low in salt and animal fats) *and* adoption of a healthy lifestyle (physical activity, no smoking, no or moderate alcohol, maintaining a lean BMI less than 25kg/m^2^).

22.	Aerobic exercise, such as brisk walking, for at least 30 minutes per day at least five days per week should be encouraged. Women should be informed that if they are able to exercise beyond the recommended level (30 minutes per day at least five days per week), the cardiometabolic benefits are even greater.

	**Screening for Cardiometabolic and Kidney Disease Risk Markers**

23.	Screening for cardiometabolic risk factors should commence at six to eight weeks postpartum (measurement of BP, BMI and fasting blood glucose).

24.	Lipid profiling (total cholesterol, HDL cholesterol, LDL cholesterol and triglycerides) should not be undertaken during the six-week postpartum screening.

25.	If feasible, the first screening schedule at six to eight weeks postpartum should be integrated with the six- to eight-week postpartum review by obstetricians/midwives or other maternity care providers, as appropriate, for continuity of care and to enhance compliance.

26.	If cardiometabolic markers are normal during the six- to eight-week postpartum screening, women should be referred to their primary care providers for continuation of follow-up and ongoing screening.

27.	If cardiometabolic markers are abnormal during the six- to eight-week postpartum screening, women should be referred to cardiologists or general physicians for continuation of follow-up and ongoing screening.

28.	Further cardiometabolic risk screening should be undertaken at six months postpartum and annually thereafter. This should include lipid profiling (measurement of BP, BMI, fasting blood glucose, total cholesterol, HDL cholesterol, LDL cholesterol, triglycerides).

29.	Women with HDP with persistent proteinuria and/or hypertension at six to eight weeks postpartum should be reassessed at three to six months postpartum. Women with ongoing proteinuria, decreased estimated glomerular filtration rate (eGFR) (<60 mL/min) or another indication of renal disease, such as abnormal urinary sediment, should be referred for a nephrology review.

	**Indicators of Abnormal Cardiometabolic Markers**

30.	Both women and their caregivers should be informed that their BMI should be maintained at ≤25 kg/m^2^.

31.	Both the women and their caregivers should be informed that lipid profiles should be maintained at <1.7 mmol/l for triglycerides and >1.29 mmol/l for HDL cholesterol.

32.	Both the women and their caregivers should be informed that BP should be <120 mm Hg for systolic BP and <80 mm Hg for diastolic BP.

33.	Both the women and their caregivers should be informed that their fasting blood glucose should be maintained at < 5.6 mmol/l or <100 mg/dl.


## Discussion

This systematic review and Delphi consensus survey resulted in 33 recommendations (in six categories) to guide the prevention and screening for cardiometabolic and kidney disorders in women with a history of HDP that are deemed compatible and feasible for deployment in LMIC settings. The high response rate of almost 80% in the first round of the Delphi survey speaks volumes of the imperative of this piece of work. Although the document is developed with LMICs in mind, these recommendations may be useful in all settings, as, in our knowledge, this is the first comprehensive and detailed guideline on this topic.

Health care workers and maternity care providers will only maximize the use of this guideline if they are able to identify HDP and its classifications correctly. Lack of global consensus in the diagnosis of preeclampsia and other HDP variants has militated against harmonized efforts in HDP management [23]. To avoid lack of clarity, we recommend universal adoption of HDP definitions and classification into preeclampsia, gestational hypertension and chronic hypertension in pregnancy, as proposed by the International Society for the Study of Hypertension in Pregnancy (ISSHP) [1].

While this guideline is intended for postpartum period use, the actual counseling and associated educational activities should start as early as possible during the antennal period once the diagnosis of HDP is established, as the antenatal period provides a better teachable moment for adoption of healthy living [24]. If, for any reason, antenatal counseling was not provided, then the next best chance and preferred timing of counseling is the immediate postpartum period and certainly before discharge [25].

Implementation of this guideline should not be restricted to only obstetricians/gynecologists, midwives and other medical specialists. In LMICs, many pregnant women are attended to by cadres of health care providers without specialized skills in obstetrics and midwifery [26]. Therefore, we recommend that all health care cadres working in settings where pregnant women attend prenatal and intrapartum care (for example, community health workers or general medical practitioners) should be trained and mentored in the use of this guideline, as this will substantially reduce adverse short- and long-term health outcomes. The counseling and preventive services can be embedded within the existing health system’s infrastructure as routine postpartum or newborn clinics. In addition, given the high risk of women with severe and/or early HDP for future noncommunicable disease risks [2,3,10,11,12,13,14,15], a dedicated postpartum clinic to cater for women with HDP could be instituted where resources permit. In addition, evidence from patients’ group perspectives suggests that (some of) these services could also be delivered online, via a mobile device or via peer-to-peer support in a safe community of shared experience [27,28].

While this guideline is specifically tailored to women with prior HDP, it should be emphasized that for women with nontraditional cardiometabolic risk factors, such as those with intrauterine growth restriction, gestational diabetes, abruptio placentae and preterm delivery, these interventions may be beneficial as well and should be considered in all the counseling/health education activities and follow-up [27]. This is not surprising because HDP (especially preeclampsia) and the so-called nontraditional obstetrics cardiovascular disease (CVD) risk factors have shared pathogenesis mechanisms: the maternal placental syndromes [29]. Given the concurrent rise of both HDP as well as cardiometabolic and renal diseases globally, an inclusive and harmonized effort to target women most at risk will be beneficial.

While there are divergent views, and in some instances lack of clarity [30,31], on the right timing of initiation of screening for cardiometabolic risk markers, our panel aligned with an increasing body of opinions to initiate this at six to eight weeks postpartum [9,13,27,32]. From six weeks postpartum, screening of risk markers (including hypertension, proteinuria and BMI) could commence. However, lipid profiling should not commence at six weeks postpartum until later dates due to substantial variability in serum lipids at this time [7].

This guidance utilizes mixed methods designs comprising initial systematic review followed by a standard Delphi survey process, ensuring that evidence derived from global practices can be used to improve care for women with HDP in LMIC settings. And because of its comprehensive, detailed and systematic presentation, it could supplement local and regional guidelines, even in HICs, given the lack of evidence on the impact of implementation of existing guidelines in those settings. In addition, future implementation studies will be necessary to evaluate barriers and opportunities for integration within existing health systems and its impact on health outcomes of women. On the other hand, this guideline is weakened by a relatively poor representation of midwives (14.3%) in the final Delphi survey as compared to obstetricians (61.6%). However, this is not expected to affect its validity, as the 10 midwives surveyed were deemed adequate to provide opinions on the feasibility of its implementation.

## Conclusion

These guiding recommendations for health workers in LMICs are intended to provide simple, accessible, practicable and highly effective preventive and screening approaches for cardiovascular diseases, metabolic syndrome and chronic kidney diseases in women with prior HDP. As this is the first such detailed and comprehensive work in this field, the guideline may also be useful for practitioners in HICs. In addition, implementation of these recommendations will provide a yardstick for future evaluation and validation of the 33 guiding recommendations and consequently reduce the rising burden of disability and mortality from chronic noncommunicable diseases in LMICs.

## Data Accessibility Statement

All data related to this manuscript can be obtained from the first author on request.

## Additional File

The additional file for this article can be found as follows:

10.5334/gh.1195.s1 Appendixes. Appendix I and III.
