# Supplementary material for: Prevention and Screening for Cardiometabolic Disease Following Hypertensive Disorders in Pregnancy in Low-Resource Settings: A Systematic Review and Delphi Study
Source: Glob Heart. 2023 Apr 25;18(1):21. doi: 10.5334/gh.1195 (PMC10143945; doi:10.5334/gh.1195)
Supplement: Appendixes. — Appendix I and III. [file gh-18-1-1195-s1.pdf]

Appendixes

Appendix I: Search strings used on PubMed which was slightly modified to suit other electronic databases:

(((Chronic medical disorder*[Mesh] OR Cardiovascular diseas*[Mesh] OR Metabolic syndrome [Mesh] OR Chronic kidney diseas*[Mesh] OR Chronic renal diseas*[Mesh] OR End-staged kidney diseas*[Mesh] OR End-stage renal diseas*[Mesh] AND Hypertensive disorders in pregnancy {Mesh] OR Pregnancy-induced hypertension [Mesh] OR Pre-eclampsia [Mesh] OR Eclampsia [Mesh] OR Gestational hypertension [Mesh] AND Guidelines [Title/Abstract]))).

Appendix II: Delphi survey questionnaire based on the thirty-five guiding recommendations summarized from the systematic review process

1. The following pertains to standard definitions of how hypertensive disorders in pregnancy should be identified. It is important that we achieve a unified consensus in its identification for effective management. For each of the following definitions of HDP, indicate your level of agreement with each of the definition and classifications (i.e strongly disagree, disagree, neutral, agree or strongly agree)

	**Strongly disagree**	**Disagree**	**Neutral**	**Agree**	**Strongly agree**
Identifying pregnant women with hypertensive disorders in pregnancy					
1.1. As recommended by the International Society for the Study of Hypertension in Pregnancy, HDP should be classified as gestational hypertension, chronic hypertension in pregnancy and pre-eclampsia					
Any comment?					
1.2. Chronic hypertension in pregnancy should be diagnosed as any hypertension with onset before the index pregnancy or diagnosed within the first 20 weeks of the index pregnancy					
Any comments?					
1.3. Gestational hypertension should be diagnosed as any hypertension occurring after the first 20 weeks of pregnancy without significant proteinuria (<2++ of proteinuria on urine dipstick measurement) or any hematological or biochemical abnormality					
Any comment?					
	**Strongly disagree**	**Disagree**	**Neutral**	**Agree**	**Strongly agree**
1.4. Preeclampsia should be diagnosed as hypertension with onset after the first 20 weeks of pregnancy with significant proteinuria (≥2++ of proteinuria on urine					

dipstick measurement) or the presence of any hematological and biochemical abnormality					
Any comment?					
2. Timing of first counseling/health education<br>The following two statements pertain to what should be the preferred timing for counseling women with HDP on their risks of future cardiometabolic and kidney diseases. For each of the statement, indicate your level of agreement with the recommended timeline (i.e strongly disagree, disagree, neutral, agree or strongly disagree)					
2.1. Counselling on cardiometabolic risk following HDP should start early in pregnancy as this period provides a better teachable moment for adoption of healthy living					
Any comment?					
2.2. If counselling was not provided during the pregnancy, the next best opportunities should be either in the immediate postpartum period before discharge OR during the 2 weeks postpartum review					
Any comment?					
3.0 Structure/Setting of care<br>The following statements pertain to optimal setting or structure in which risk counseling services should take place for women with HDP on their risks of future cardiometabolic and kidney diseases. For each of the statements, indicate your level of agreement with the recommended setting/structure (i.e strongly disagree, disagree, neutral, agree or strongly agree)					
3.1. Counseling should be performed at facilities that women can access and by any available trained health care provider regardless of their specialties					
Any comment?					
**3.2**. Postpartum care counseling should be delivered within a trauma-informed model as women with post-traumatic experience are less likely to return to health facilities for regular monitoring					

Any comment?					
**3.3**. Where feasible, women with hypertensive disorders in pregnancy should be reviewed within a multi-disciplinary clinic involving Obstetricians/Midwives, primary care physicians, cardiologists and mental health experts to reduce inequities in health					
Any comment?					
**3.4**. Obstetricians, midwives, and maternity care providers should routinely counsel women with hypertensive disorders in pregnancy on their risk for cardiometabolic and kidney disorders					
Any comment?					
**3.5**. Where practicable, a dedicated postpartum clinic for hypertensive disorders in pregnancy be established to facilitate transition of care and to provide window of opportunities to focus on improving cardiometabolic health, primary prevention of CVD and counselling on risk factors modification					
Any comment?					
**3.6**. Adopt inclusion and utilization of best practice alerts in electronic medical records to facilitate risks identification and improve follow up					
Any comment?					
	**Strongly disagree**	**Disagree**	**Neutral**	**Agree**	**Strongly agree**

**3.7**. All maternity centers should formulate a dedicated guideline for women with hypertensive disorders in pregnancy for their continuity of care from Obstetricians/Midwives, primary care physicians or specialists as appropriate					
Any comment?					
**3.8**. All maternity centers should develop a comprehensive pregnancy history tool for CVD risk assessment to enable elucidation of non-traditional CVD risk factors (for example, gestational diabetic, intra-uterine growth restriction and preterm delivery)					
Any comment?					
**3.9**. Women with other non-traditional risk factor for cardiometabolic diseases such as gestational diabetes, intra-uterine growth restriction and preterm delivery should also be counselled and monitored postpartum					
Any comment?					
**3.10**. Where feasible, antenatal care card/folder/record should be modified to include section on documentation of postpartum risk assessment and monitoring of long-term risks of chronic medical conditions associated HDP and other pregnancy complications					
Any comment?					
3.11. All health care providers of maternity services should be trained on the links between hypertensive disorders in pregnancy, cardiometabolic and chronic kidney disorders					
Any comment?					
**3.12**. An health care provider checklist should be provided as working tool to ensure detailed and balanced communication of cardio-metabolic disease risks to patients with hypertensive disorders in pregnancy					

Any comment					
	**Strongly disagree**	**Disagree**	**Neutral**	**Agree**	**Strongly agree**
4.0 Counselling information needs for women identified with HDP<br>The following statements pertain to the kind of counseling information for women with HDP on their risks of future cardiometabolic and kidney diseases. For each of the statements, indicate your level of agreement with the recommended counselling information to be given to these women (i.e strongly disagree, disagree, neutral, agree or strongly agree))					
**4.1**. All women with HDP should be informed of their increased risk of cardiometabolic and chronic kidney diseases in later life					
Any comment?					
**4.2**. Counselling women on behavior modification should express the risk of cardio-metabolic disorders as probability scores, expressed as chances (%) of developing the disease condition					
Any comment?					
**4.3**. Women with HDP (especially women who are overweight – BMI $\geq 25 kg/m^2$) should be informed that postpartum lifestyles modification, as the first approach, substantially reduces the risk of cardiometabolic diseases in later life					
Any comment?					
**4.4**. Lifestyle modification should include adopting a healthy diet (all or any combination of consumption of fruits, vegetables, plant protein and oily fish **AND** reduction or combination of any of diets low in salt and animal fats) **AND** adoption of healthy lifestyles (physical activities, no smoking, no or moderate alcohol, maintaining a lean body mass index less than $25 kg/m^2$)					
Any comment?					

**4.5.** Aerobic exercise such as brisk walking for, at least, 30 minutes per day for, at least 5 days per week should be encouraged. Women should be informed that if they are able to exercise beyond the recommended level (30 minutes per day for, at least 5 days per week), the cardiometabolic benefits are even greater					
Any comment?					
	**Strongly disagree**	**Disagree**	**Neutral**	**Agree**	**Strongly agree**
5.0. Screening for cardiometabolic and kidney disease risk markers: timing and approach<br>The following statements pertain to optimal timing and approach for assessing cardiometabolic risk markers in women following HDP. For each of the statements, indicate your level of agreement with the recommended timing and/or approach (i.e strongly disagree, disagree, neutral, agree or strongly agree)					
**5.1**. Screening for cardiometabolic risk factors should commence at between 6 – 8 weeks postpartum (measurement of blood pressure, BMI and fasting blood glucose).					
Any comment?					
**5.2**. Lipid's profiling (total cholesterol, HDL cholesterol, LDL cholesterol and triglycerides) should not be undertaken during the 6 – weeks postpartum screening as there is substantial change at this time.					
Any comment?					
**5.3**. If feasible, the first screening schedule at between 6 – 8 weeks postpartum should be integrated with the 6 -8 weeks postpartum review by Obstetricians, Midwives, or other maternity care providers, as appropriate, for continuity of care and to enhance compliance					
Any comment?					

**5.4**. If cardiometabolic markers are normal during the 6 – 8 postpartum screening, women should be referred to their primary care providers for continuation of follow up and ongoing screening					
Any comment?					
**5.5.** If cardiometabolic markers are abnormal during the 6 – 8 postpartum screening, women should be referred to cardiologists or general physicians for continuation of follow up and ongoing screening					
Any comment?					
**5.6**. Women with hypertensive disorders in pregnancy should have their urine protein (dipstick measurement or 24-hour urine protein estimation) estimated at between 6 – 8 weeks postpartum. If there is no proteinuria or hypertension during this review, no further follow up is necessary					
Any comment?					
	**Strongly disagree**	**Disagree**	**Neutral**	**Agree**	**Strongly agree**
**5.7**. Further cardiometabolic risk screening should be undertaken at 6 months postpartum and annually thereafter. This should include lipids profiling (measurement of blood pressure, BMI, fasting blood glucose, total cholesterol, HDL cholesterol, LDL cholesterol, triglycerides)					
Any comment?					
**5.8.** Women with hypertensive disorders in pregnancy with persistent proteinuria and/or hypertension at 6-8 weeks postpartum should be re-assessed at between 3 – 6 months postpartum. Women with ongoing proteinuria, decreased estimated glomerular filtration rate (eGFR) (< 60 mL/min), or another indication of renal disease, such as abnormal urinary sediment should be referred for a nephrology review					
Any comment?					

6.0 Target indicators of abnormal cardiometabolic markers women should be informed of?<br>The following statements pertain to what screening indicator(s) should be targeted and communicated in the assessment of women with HDP on their risks of future cardiometabolic and kidney diseases. For each of the statements, indicate your level of agreement with the recommended thresholds (i.e strongly disagree, disagree, neutral, agree or strongly agree)					
**6.1**. Both the women and their caregivers should be informed that their body mass index should be maintained at ≤25kg/m^2					
Any comment?					
**6.2**. Both the women and their caregivers should be informed that lipids profiles should maintained at < 1.7mmol/l for triglycerides and < 1.29mmol/l for high density lipoproteins cholesterol					
Any comment?					
**6.3.** Both the women and their caregivers should be informed that blood pressure should be at ≤130mmHg for systolic blood pressure and ≤85mmHg for diastolic blood pressure					
Any comment?					
**6.4**. Both the women and their caregivers should be informed that their fasting blood glucose should be maintained at < 5.6mmol/l or <100mg/dl					
Any comment?					

Appendix III: Summary of consensus for the various outcomes (i.e., in/out/no consensus).

Round on inclusion when consensus was reached	Round on exclusion when consensus was reached
Identifying women with HDP	
As recommended by the ISSHP, HDP should be classified as Chronic hypertension in pregnancy, gestational hypertension and pre-eclampsia (round 1)	
Chronic hypertension in pregnancy should be diagnosed as any hypertension with onset before the index pregnancy or diagnosed within the first 20 weeks of the index pregnancy (round 2)	
Gestational hypertension should be diagnosed as any hypertension occurring after the first 20 weeks of pregnancy without significant proteinuria (<2++ of proteinuria on urine dipstick measurement) or any hematological or biochemical abnormality (round 2)	
Preeclampsia should be diagnosed as hypertension with onset after the first 20 weeks of pregnancy with significant proteinuria (≥2++ of proteinuria on urine dipstick measurement) or the presence of any hematological and biochemical abnormality (round 2)	
Timing of first counseling/health education	
Counseling on cardiometabolic risk following HDP should start early in pregnancy as this period provides a better teachable moment for adoption of healthy living (round 1)	
If counselling was not provided during the pregnancy, the next best opportunities should be either in the immediate postpartum period before discharge OR during the 2 weeks postpartum review (round 1)	
Structure and setting of care	
Counseling should be performed at facilities that women can access and by any available trained health care provider regardless of their specialties (round 1)	Postpartum care counseling should be delivered within a trauma-informed model as women with post-traumatic experience are less likely to return to health facilities for regular monitoring (round 1)
Where feasible, women with hypertensive disorders in pregnancy should be reviewed within a multi-disciplinary clinic involving Obstetricians/Midwives, primary care physicians, cardiologists and mental health experts to reduce inequities in health (round 1)	

Obstetricians, midwives, and maternity care providers should routinely counsel women with hypertensive disorders in pregnancy on their risk for cardiometabolic and kidney disorders (round 1)	
Where practicable, a dedicated postpartum clinic for hypertensive disorders in pregnancy be established to facilitate transition of care and to provide window of opportunities to focus on improving cardiometabolic health, primary prevention of CVD and counselling on risk factors modification (round 1)	
Adopt inclusion and utilization of best practice alerts in electronic medical records to facilitate risks identification and improve follow up (round 1)	
All maternity centers should formulate a dedicated guideline for women with hypertensive disorders in pregnancy for their continuity of care from Obstetricians/Midwives, primary care physicians or specialists as appropriate (round 1)	
All maternity centers should develop a comprehensive pregnancy history tool for CVD risk assessment to enable elucidation of non-traditional CVD risk factors (for example, gestational diabetic, intra-uterine growth restriction and preterm delivery) (round 1)	
Women with other non-traditional risk factor for cardiometabolic diseases such as gestational diabetes, intra-uterine growth restriction and preterm delivery should also be counseled and monitored postpartum (round 1)	
Where feasible, antenatal care card/folder/record should be modified to include section on documentation of postpartum risk assessment and monitoring of long-term risks of chronic medical conditions associated HDP and other pregnancy complications (round 1)	
All health care providers of maternity services should be trained on the links between hypertensive disorders in pregnancy, cardiometabolic and chronic kidney disorders (round 1)	
A health care provider checklist should be provided as working tool to ensure detailed and balanced communication of cardio-metabolic disease risks to patients with hypertensive disorders in pregnancy (round 1)	
Counseling information needs for women identified with HDP	
All women with HDP should be informed of their increased risk of cardiometabolic and chronic kidney diseases in later life (round 1)	

Counseling women on behavior modification should express the risk of cardio-metabolic disorders as probability scores, expressed as chances (%) of developing the disease condition (round 1)	
Women with HDP (especially women who are overweight – BMI ≥25kg/m2) should be informed that postpartum lifestyles modification, as the first approach, substantially reduces the risk of cardiometabolic diseases in later life (round 2)	
. Lifestyle modification should include adopting a healthy diet (all or any combination of consumption of fruits, vegetables, plant protein and oily fish AND reduction or combination of any of diets low in salt and animal fats) AND adoption of healthy lifestyles (physical activities, no smoking, no or moderate alcohol, maintaining a lean body mass index less than 25kg/m2) (round 2)	
Aerobic exercise such as brisk walking for, at least, 30 minutes per day for, at least 5 days per week should be encouraged. Women should be informed that if they are able to exercise beyond the recommended level (30 minutes per day for, at least 5 days per week), the cardiometabolic benefits are even greater (round 2)	
Screening for cardiometabolic and kidney disease risk markers	
Screening for cardiometabolic risk factors should commence at between 6 – 8 weeks postpartum (measurement of blood pressure, BMI and fasting blood glucose) (round 1)	
Lipid's profiling (total cholesterol, HDL cholesterol, LDL cholesterol and triglycerides) should not be undertaken during the 6 – weeks postpartum screening as there is substantial change at this time (round 1)	
If feasible, the first screening schedule at between 6 – 8 weeks postpartum should be integrated with the 6 -8 weeks postpartum review by Obstetricians, Midwives, or other maternity care providers, as appropriate, for continuity of care and to enhance compliance (round 1)	
If cardiometabolic markers are normal during the 6 – 8 postpartum screening, women should be referred to their primary care providers for continuation of follow up and ongoing screening (round 2)	
If cardiometabolic markers are abnormal during the 6 – 8 postpartum screening, women should be referred to cardiologists or general physicians for continuation of follow up and ongoing screening (round 1)	

	Women with hypertensive disorders in pregnancy should have their urine protein (dipstick measurement or 24-hour urine protein estimation) estimated at between 6 – 8 weeks postpartum. If there is no proteinuria or hypertension during this review, no further follow up is necessary (round 1)
Further cardiometabolic risk screening should be undertaken at 6 months postpartum and annually thereafter. This should include lipids profiling (measurement of blood pressure, BMI, fasting blood glucose, total cholesterol, HDL cholesterol, LDL cholesterol, triglycerides) (round 1)	
Women with hypertensive disorders in pregnancy with persistent proteinuria and/or hypertension at 6-8 weeks postpartum should be re-assessed at between 3 – 6 months postpartum. Women with ongoing proteinuria, decreased estimated glomerular filtration rate (eGFR) (< 60 mL/min), or another indication of renal disease, such as abnormal urinary sediment should be referred for a nephrology review (round 1)	
Indicators of abnormal cardiometabolic markers	
Both women and their caregivers should be informed that their BMI should be maintained at ≤25kg/m^2 (round 1)	
Both the women and their caregivers should be informed that lipids profiles should be maintained at < 1.7mmol/l for triglycerides and < 1.29mmol/l for high density lipoproteins cholesterol (round 1)	
Both the women and their caregivers should be informed that blood pressure should be <120mmHg for systolic blood pressure and <80mmHg for diastolic blood pressure (round 1)	
Both the women and their caregivers should be informed that their fasting blood glucose should be maintained at < 5.6mmol/l or <100mg/dl (round 1)	
